# Adolescent Afghan Refugees Display a High Prevalence of Hyperhomocysteinemia and Associated Micronutrients Deficiencies Indicating an Enhanced Risk of Cardiovascular Disease in Later Life

**DOI:** 10.3390/nu14091751

**Published:** 2022-04-22

**Authors:** Muhammad Shabir Khan, Anum Saeedullah, Simon C. Andrews, Khalid Iqbal, Syed Abdul Qadir, Babar Shahzad, Zahoor Ahmed, Muhammad Shahzad

**Affiliations:** 1 Institute of Basic Medical Sciences, Khyber Medical University, Hayat Abad Phase 5, Peshawar 25120, Pakistan; mshabbir.kmu@gmail.com (M.S.K.); khalid.ibms@kmu.edu.pk (K.I.); qadirmasoomi@gmail.com (S.A.Q.); babar.kmu@gmail.com (B.S.); 2 Department of Biochemistry, Kabir Medical College, Gandhara University, Canal Road University Town, Peshawar 25000, Pakistan; anumsaeedullah1990@gmail.com; 3 School of Biological Sciences, Health and Life Sciences Building, University of Reading, Reading RG6 6AX, UK; 4 Department of Biochemistry, Khyber Medical College, Peshawar 25120, Pakistan; zakhan70@yahoo.co.uk

**Keywords:** malnutrition, homocysteine, refugees, vulnerable, vitamin B12, folate

## Abstract

A growing body of research evidence suggests that elevated homocysteine level (hyperhomocysteinemia) is an independent risk factor for cardiovascular diseases. The current study aimed to investigate the prevalence and associated risk factors for hyperhomocysteinemia among adolescent Afghan refugees aged 10–19 years. In total, 206 healthy adolescent boys and girls were randomly recruited from a refugee village in Peshawar, Pakistan, in 2020. Socio-demographic data, anthropometric assessment, and blood sample collection were performed following standard methods. Serum homocysteine was assessed using a chemiluminescent microparticle immunoassay, with hyperhomocysteinemia defined as levels ≥ 15 µmol/L. The overall prevalence of hyperhomocysteinemia was 25%, with mean homocysteine levels significantly (*p* = 0.004) higher among boys (14.1 µmol/L) than girls (11.8 µmol/L). Multivariate logistic regression analysis revealed a significant association between hyperhomocysteineimia and serum levels of vitamin B12 (OR 0.29; 95% CI of 0.14 to 0.62; *p* < 0.01) and folate (OR 0.1; 95% CI of 0.03 to 0.27; *p* < 0.001). Overall, our study findings indicate high prevalence of hyperhomocysteinemia among adolescent Afghan refugees who are potentially at high risk of developing cardiovascular diseases in future. There is a dire need to develop and implement nutritional and public health strategies to control hyperhomocysteinemia, protect against related diseases and complications in future, and ensure healthy lives and well-being among these vulnerable populations.

## 1. Introduction

According to latest report (2021) of the United Nations Higher Commission for Refugees (UNHCR), around 84 million people across the world are forcibly displaced from their homeland, of which 26.2 million were refugees. They are people who were forced to flee their home country due to war, violence, conflict, or well-founded fear of persecution and crossed an international border and took refuge in another country [1]. Around 68% of these refugees have originated from just five countries, with Afghanistan ranking third in the list after Syria and Venezuela. The majority of the Afghan refugees have taken refuge in Pakistan, which has been host to one of the largest refugee populations in the world over the last few decades. Thus, despite its deep-rooted socio-political, security, and economic issues, Pakistan currently accommodates around 2.4 million registered and non-registered Afghan refugees [1].

Afghan refugees in Pakistan suffer from deficient housing, food, social services, and health. One of the most serious issues that refugees frequently face is the lack of access to an affordable and effective healthcare system when occupying a developing country such as Pakistan [2]. A recent report on disease status among Afghan refugees in Pakistan reported a high prevalence of communicable and non-communicable diseases [3]. Cardiovascular disease (CVD) has been found to be the main cause of mortality, resulting in around 25% of deaths among Afghan refugees although CVD is also common and a leading cause of death in the general Pakistan population [4].

CVD is a major public health concern across the world. It is not only the leading cause of death and disability but also poses a significant burden on national public health systems. Risk factors for CVD include (but are not limited to) age, gender, dyslipidemia, obesity, sedentary life style, and genetic factors [5]. A further significant contributory factor is elevated levels of homocysteine in blood (hyperhomocysteinemia); indeed, this is increasingly being recognized as an important factor in the development of atherosclerosis [6].

Homocysteine is a non-protein-forming sulfur-containing amino acid and an intermediate product in methionine metabolism [7]. Normally, homocysteine concentration in blood is very low due to cellular homocysteine export activity [8]. However, genetic defects in the gene encoding enzymes involved in homocysteine metabolism or depletion of important cofactors (vitamin B12, vitamin B6, and folate) required for homocysteine metabolism may result in elevated levels of homocysteine [9]. McCully and colleagues (1968) were the first to propose the homocysteine theory of atherosclerosis, whereby an increase in homocysteine, even if moderate, greatly accelerates the development of atherosclerosis in humans [10]. Subsequent research has shown a strong association between elevated homocysteine levels and increased risk of developing cardio-metabolic disorders, including stroke, hypertension, ischemic heart diseases, deep vein thrombosis, and heart failure [11,12,13,14,15]. Indeed, systematic reviews and meta-analysis studies suggest that with every 5 µmol/L increase in serum homocysteine levels, there is 20% increased risk of developing CVD and associated complications [16,17]. Therefore, maintaining a normal blood level of homocysteine is crucial for lowering the risk of developing CVD. For instance, reducing homocysteine levels by just 3 µmol/L would reduce the risk of ischemic heart disease, deep vein thrombosis, and stroke by 16, 25, and 24%, respectively [16].

Afghan refugees in Pakistan constitute a vulnerable population group facing numerous health-related problems. This is illustrated by our recent report of widespread malnutrition among adolescent Afghan refugees residing in Peshawar, Pakistan [18], which included multiple micronutrients deficiencies, including vitamin D, vitamin B12, and folate. Since deficiency of folate and vitamin B12 causes susceptibility to raised homocysteine, we also explored the prevalence of hyperhomocysteinemia and associated factors among adolescent Afghan refugees in Pakistan to gain an impression of any raised risk of the development of atherosclerosis.

## 2. Materials and Methods

### 2.1. Study Participants and Data Collection

This population-based, cross-sectional study was conducted in Khazana refugee camp Peshawar, Pakistan, in March–April 2020. Details for the study are provided in our recently published manuscript [18]. A total of 206 healthy adolescents were recruited, both boys and girls, aged 10–19 years and living in the same locality for at least one year. Non-consenting individuals, those suffering from chronic illness currently or in the recent past, and pregnant or lactating women were excluded from the study. Before data and sample collection, all participants and their parents or legal guardians were provided with study information sheets in their local language (Pashto and Dari), which was followed by obtaining written informed consent. The participants were also briefed verbally by the trained data collectors about the study aims and objectives and the steps involved. Demographic and socioeconomic data from the participants were collected using a structured questionnaire consisting of 19 open- and close-ended questions regarding demographic characteristics such as age, gender, education, and socioeconomic status. The study was approved by the Ethics Board of Khyber Medical University. Administrative approval of the study was also obtained from Commissionerate Afghan Refugees, Khyber Pakhtunkhwa, Pakistan.

### 2.2. Anthropometric Measurements

Weight and height of all participants were assessed before sample collection by trained data collectors. A wall-mounted stadiometer and calibrated digital balance (Seca Ltd., Birmingham, UK) were used to record height and weight to the nearest 0.5 cm and 0.1 kg, respectively, following standard procedures. The measurements were recorded three times and an average taken. Body mass index (BMI) was calculated by dividing body weight (kg) by height (m^2^).

### 2.3. Collection of Blood Samples and Laboratory Analysis

Peripheral blood specimens (non-fasting) were collected from all participants by a trained phlebotomist using vacuum syringes (BD Diagnostics, Basel, Switzerland). Complete blood counts (CBC) were performed on whole blood using a Sysmex automatic hematology analyzer (XP-100, Jalan Tukang, Singapore). Blood samples were centrifuged to separate plasma and serum and stored at −80 °C till further analysis for ferritin, vitamin B12, folate, and vitamin D assay. Serum homocysteine was assessed using a chemiluminescent microparticle immunoassay (CMIA) assay using an ARCHITECT i2000 analyzer (Abbott Diagnostics, Zug, Switzerland). The assay is based on the reduction of bound or dimerized homocysteine (oxidized form) to free homocysteine through the action of dithiothreitol (DTT). In the presence of excess adenosine, the recombinant enzyme S-adenosyl homocysteine hydrolase (rSAHHase) coverts free homocysteine to S-adenosyl homocysteine (SAH). The SAH then competes with acridinium-labeled S-adenosyl cysteine for a particle-bound monoclonal antibody resulting in chemiluminescence, which is measured as relative light units (RLUs) via ARCHITECToptics. Serum homocysteine levels ≥15 µmol/L were considered as indicative of hyperhomocysteinemia [19].

### 2.4. Statistical Analysis

Numerical variables including age, BMI, and biomarkers are presented as median and inter-quartile range values and geometric means (95% confidence intervals). Categorical variables including obesity, income quartiles, educational status, family size, and gender are described as frequencies (percentages). Homocysteine observations were log-transformed prior to subsequent analysis. For continuous and categorical variables, *t*-test and chi-square tests were used, respectively, to investigate differences between male and female study participants. Concentration response for homocysteine levels with age, BMI, educational status, income status, family size, serum ferritin, serum folate, and vitamin B12 were assessed using linear regression. For this analysis, Zscore of homocysteine was used for the association. Logistic regression was used to investigate association of homocystenemia with potential risk factors. Biomarkers were log-transformed before analysis. Assumptions of logistic regression were assessed using residual plots. All analysis were performed in SAS (Version 9.4, SAS Institute Inc., Cary, NC, USA).

## 3. Results

Of the total study population (*n* = 206), homocysteine data could only be collected for 190 participants. Background characteristics of these study participants (*n* = 190) are described in Table 1. Median age of the participants was 13 years with no significant difference between adolescent boys and girls. The majority (*n* = 121; 63.7%) of the participants were 10–14 years old. Only 11.2% of the participants were obese, with no significant difference in obesity levels between boys and girls. Around 80% of the participants either had no formal education or studied only to primary level. The average family size was around 12.

Table 2 presents the percentile distribution of homocysteine levels by gender and age categories. Overall, the mean plasma homocysteine concentration ranged from 4.5 to 37.3 µmol/L. The median level of homocysteine was 12.2 µmol/L, with 25% of the subjects categorized as having hyperhomocysteinemia (homocysteine levels ≥15 µmol/L).

Mean homocysteine levels were significantly higher among boys as compared to girls. However, no significant differences were observed between the two age categories. Distribution of homocysteine levels for both the genders are shown in Supplementary Figure S1.

**Figure 1:**
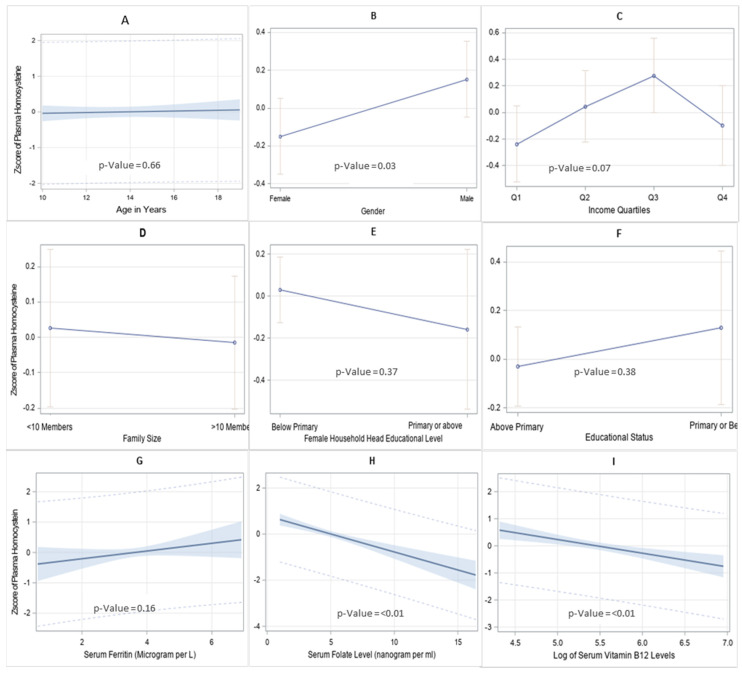
Associations between plasma homocysteine levels. Associations are shown for plasma homocysteine Z-score and age (**A**), gender (**B**), income (**C**), family size (**D**), educational status of the female household head (**E**), education level of the participant (**F**), serum ferritin (**G**), folate (**H**), and vitamin B12 (**I**). Means and 95% confidence limits are presented for gender and income quartiles from *t*-test and ANOVA, respectively. For age, serum ferritin, folate, and B12, regression lines with 95% confidence interval are shown, while for gender, income quartiles, family size, educational level of female household, and educational status of the respondents, mean Zscore of plasma homocysteine for the groups with 95% confidence intervals are shown.

The relationship between plasma homocysteine levels and associated factors, as assessed by concentration-response curve based on Z-score, is presented in Figure 1. While age has no effect on homocysteine levels among adolescents (Figure 1A), significant difference was observed for gender, with male participants displaying higher homocysteine levels than females (Figure 1B). Similarly, the Z-scores for plasma homocysteine were affected by income (Figure 1C), family size (Figure 1D), education level of both the participants (Figure 1E) and the female head of the household (Figure 1F), and serum ferritin levels (Figure 1G); however, none of these differences (Figure 1B–G) were significant. In contrast, plasma homocysteine levels decrease significantly with increasing folate (Figure 1H) and vitamin B12 levels (Figure 1I).

Logistic regression analysis, both uni-variable and multivariable, of risk factors for hyperhomocysteinemia is presented in Table 3. No significant associations were observed between hyperhomocysteinemia and age, gender, and educational status. Participants from small families (<10 household members) had higher odds (OR 3.64; 95% CI of 1.56 to 8.49; *p* < 0.01) of homocysteinemia as compared to large families. A significantly lower levels of serum vitamin B12 and folate were observed among participants with hyperhomocysteinemia (homocysteine levels ≥ 15 μmol/L). Multivariate logistic regression analysis revealed a statistically non-significant positive association between serum homocysteine and serum ferritin (OR 1.55, 95% CI of 0.93 to 2.57; *p* = 0.09), whereas a strong inverse association of homocysteineimia was observed with serum vitamin B12 (OR 0.29; 95% CI of 0.14 to 0.62; *p* < 0.01) and folate (OR 0.1; 95% CI of 0.03 to 0.27; *p* < 0.001).

## 4. Discussion

Elevated levels of homocysteine and/or hyperhomocysteinemia have increasingly been shown to play a crucial role in different pathological conditions in humans, especially for cardiovascular, neurodegenerative, and psychiatric disorders [20,21]. Although the etiology of hyperhomocysteinemia is multifaceted, the condition is primarily caused by deficiency of folate and related B vitamins (vitamin B2, B6, and B12) that act as cofactors in homocysteine metabolism [22]. Although a number of epidemiological studies have reported prevalence of hyperhomocysteinemia in children and adolescents, the evidence in refugee population is scanty. The current study, to the best of our knowledge, is the first to report the prevalence of hyperhomocysteinemia and to examine the impact of socio-demographic and biochemical markers on in a representative sample of adolescent Afghan refugees residing in Pakistan.

Our study reports high prevalence of hyperhomocysteinemia in the subject population based on a ≥15μmol/L threshold. It is estimated that around 5–10% of the world adult population have higher than normal homocysteine levels and are at high risk of developing cardiovascular diseases especially when other risk factors are also present [23]. However, data about homocysteine levels in other age groups, such as children and adolescents, are scarce despite the fact that these populations are vulnerable due to higher nutritional requirements for effective growth and development. The available published literature indicate that hyperhomocysteinemia is relatively rare in children and adolescent populations, particularly in high-income European countries [24,25] and the USA [26,27]. The observed differences may be partially attributed to the increased consumption of animal-source foods (red meat, sea foods, dairy products, and eggs) containing high levels of vitamin B12, the most important cofactor in homocysteine metabolism. In low-income and developing countries, >20% of body energy requirements are met through consumption of animal-source foods, whereas in wealthier nations ~40% of the body energy requirements are derived from such sources [28]. The situation may be exacerbated in vulnerable population, such as refugees, whose consumption of animal-source foods is further reduced due to lower socioeconomic status, greater unemployment, and larger family size [29]. Another contributing factor for the observed differences may be high consumption of fresh fruits and vegetable vitamins in children and adolescent from high-income countries. Fruits and vegetables are rich sources of folate and other B vitamins, and increase consumption is associated with high plasma levels of these vitamins and lower homocysteine levels [21].

An age-related difference in hyperhomocysteinemia, similar to those reported previously [30,31], was observed although the differences were not significant. Similarly, gender differences were also observed, whereby boys had higher mean homocysteine (14.01 μmol/L) levels than girls (11.83 μmol/L). Although the exact mechanism underlying gender differences in homocysteine levels is not known, it is generally believed that testosterone-dependent expression of renal cystathionine beta-synthase may contribute towards gender-based differences in homocysteine levels [32]. Another possible mechanism might be differences in the rate of homocysteine remethylation, the rate of which is higher in women, resulting in lower homocysteine levels [33]. A positive association was also observed between BMI categories and hyperhomocysteinemia, as reported previously [31,34]. However, education and socioeconomic status (monthly household income) was not associated with risk of hyperhomocysteinemia. These results are also consistent with previous studies [30,35].

An inverse association between serum homocysteine concentration and serum levels of folate and vitamin B12 was observed in this study, which is in concordance with previously published reports [36,37,38]. Intracellular metabolism of homocysteine, folate, and vitamin B occur through a common metabolic pathway sharing several intermediates [39]. Intracellular metabolism of homocysteine primarily involves two metabolic pathways, namely remethylation and trans-sulfuration. In remethylation, homocysteine is converted to methionine by acquisition of a methyl group with the assistance of vitamin B12 and folate acting as co-enzymes. In contrast, the trans-sulfuration pathway involves the vitamin B6-dependent condensation of homocysteine with serine to produce cystathionine, which in turn is hydrolyzed to form cysteine [33]. Multiple micronutrient deficiencies, especially folate and vitamin B12, is a common occurrence in refugee populations [18], and as a result, they are at high risk of developing hyperhomocysteinemia and related complications. The consequences of hyperhomocysteinemia are of particular concern in children and adolescents due to association with developmental delay, neurological damage, and cardiovascular disease risk in later life [21]. Deficiency of folate and vitamin B12 and resultant hyperhomocysteinemia are clearly indicated in this study and together act as a clear predicter of high prevalence of cardiovascular disease in these population, as reported recently [3].

Although the clinical manifestation and deleterious consequences of cardiovascular diseases usually occur in adult life, there is a strong evidence suggesting that these conditions start developing during childhood and adolescence [40,41]. Besides established risk factors (obesity, dyslipidemia, diabetes, and arterial hypertension), hyperhomocysteinemia has also been identified as casual risk factor for cardiovascular disease in children. Vascular diseases are responsible for over 50% of deaths in children with genetic disorders of homocysteine metabolism (homocysteinuria) [17]. Furthermore, a significant association between homocysteine concentration, Methylene Tetrahydrofolate Reductase (MTHFR) 677T gene polymorphism, and cardiovascular disease risks is also reported [42,43]. Future studies in these population should focus on MTHFR gene polymorphism and its impact on plasma homocysteine levels and metabolism. Our study findings suggest screening and diagnosis of hyperhomocysteinemia at an early age in these vulnerable populations and addressing the associated risk factors, such vitamin B and folate deficiency, in early life through cost-effective nutrition interventions in order to avoid cardiovascular complications in future.

The current study also carries some methodological limitations. First, with a cross-sectional study design, it is difficult to identify a causal relationship between homocysteine levels and associated factors. Second, due to small sample size, our study results should be cautiously applied to the general refugee and host population. Third, the self-reported measurement of socio-demographic characteristics may also introduce bias in the data due to inherent tendency of the participants to provide socially acceptable responses.

## 5. Conclusions

The current study reported a relatively high prevalence of hyperhomocysteinemia among adolescent Afghan refugees. While age and gender are non-modifiable risk factors, there is an opportunity to introduce life style and nutrition interventions to address modifiable risk factors for hyperhomocysteinemia among adolescents. These approaches may also help to reduce the long-term risk of homocysteine-related diseases and complication. Authors should discuss the results and how they can be interpreted from the perspective of previous studies and of the working hypotheses. The findings and their implications should be discussed in the broadest context possible. Future research directions may also be highlighted.

## Figures and Tables

**Table 1:** Background characteristics of the study participants *.

Variables	Categories	Male (*n* = 94)	Female (*n* = 96)	Total (*n* = 190)	*p*-Value
Age					
	Age (years) ^3^	12.5 (11.0, 15.0)	13.5 (10.5, 16.0)	13.0 (11.0, 16.0)	0.52 ^1^
	10–14 age group	66 (70.2%)	55 (57.3%)	121 (63.7%)	0.06 ^2^
	15–19 age group	28 (29.8%)	41 (42.7%)	69 (36.3%)	
Anthropometry					
	Weight (kg) ^3^	34.8 (27.4, 50.0)	40.5 (30.0, 46.0)	38.0 (28.5, 48.0)	0.75 ^1^
	Height (cm) ^3^	141.0 (131.0, 160.0)	145.0 (130.0, 151.8)	143.3 (130.2, 155.0)	0.31 ^1^
	BMI (kg/m^2^)	18.0 (15.8, 20.1)	18.6 (17.3, 20.1)	18.4 (16.4, 20.1)	0.07 ^1^
Education level of the participants					
	No formal education	9 (9.6%)	27 (28.1%)	36 (18.9%)	<0.01 ^2^
	Primary	60 (63.8%)	54 (56.3%)	114 (60.0%)	
	High school	22 (23.4%)	15 (15.6%)	37 (19.5%)	
	College and university	3 (3.2%)	0 (0.0%)	3 (1.6%)	
Education level of the female head of the household					
	No formal education	75 (79.8%)	69 (71.9%)	144 (75.8%)	0.03 ^2^
	Primary	12 (12.8%)	7 (7.3%)	19 (10.0%)	
	High school	7 (7.4%)	17 (17.7%)	24 (12.6%)	
	College and university	0 (0.0%)	3 (3.1%)	3 (1.6%)	
Total number of household members (family size)					
	1–4	5 (5.3%)	1 (1.0%)	6 (3.2%)	0.17 ^2^
	5–9	40 (42.6%)	33 (34.4%)	73 (38.4%)	
	10–19	42 (44.7%)	51 (53.1%)	93 (48.9%)	
	20 or more	7 (7.4%)	11 (11.5%)	18 (9.5%)	
Income status					
	1st Quartile	19 (20.2%)	28 (29.2%)	47 (24.7%)	0.24 ^2^
	2nd Quartile	25 (26.6%)	28 (29.2%)	53 (27.9%)	
	3rd Quartile	24 (25.5%)	24 (25.0%)	48 (25.3%)	
	4th Quartile	26 (27.7%)	16 (16.7%)	42 (22.1%)	

* Values given are frequencies (percentages); ^1^ Kruskal–Wallis *p*-value; ^2^ chi-Square *p*-value; ^3^ values given are median (Quartile 1–Quartile 3).

**Table 2:** Percentile distribution of homocysteine levels by sex and age.

Characteristics	Geometric Mean (95% CI)	Minimum	Percentile						Maximum
			5th	25th	50th	75th	95th	99th	
All	12.9 (12.1, 13.7)	4.5	6.8	9.9	12.2	15.9	28.6	36.9	37.3
Sex									
Boys	14.1 (13.0, 15.1)	6.6	8.6	10.6	13.7	18.1	27.7	37.3	37.3
Girls	11.8 (10.8, 12.9)	4.5	6.1	9.1	11.4	14.8	30.1	36.3	36.3
*p*-Value	0.004								
Age Categories (years)									
10–14	12.8 (12.0, 13.7)	6.2	7.9	9.6	12.5	15.5	25.4	30.1	35.5
15–19	12.9 (11.5, 14.6)	4.5	5.9	10.1	11.8	16.2	32.6	37.3	37.27
*p*-Value	0.87								

**Table 3:** Univariate and multivariate analysis of risk factors and biomarkers for hyperhomocysteinemia.

Variables	Categories	Homocysteine Levels (Freqeuncy/Median (IQR)			Uni-Variable Logistic Regression		Multivariable Logistic Regression	
		<15 μmol/L	≥15 μmol/L	*p*-Value	OR (95% CI)	*p*-Value	Adjusted	*p*-Value
Age categories (years)	10–14	86 (64.2%)	35 (62.5%)	0.83	0.91 (0.48, 1.74)	0.77	0.86 (0.3, 2.42)	0.77
	15–19	48 (35.8%)	21 (37.5%)		Ref.		Ref.	
Gender	Male	11.3 (9.6, 13.6)	20.1 (16.7, 25.2)	<0.01	1.92 (1.03, 3.64)	0.04	1.19 (0.43, 3.27)	0.74
	Female	10.3 (8.3, 11.9)	20.3 (16.9, 28.6)	<0.01				
BMI (kg/m^2^)		18.3 (16.7, 20.1)	18.4 (15.4, 20.2)	0.4	0.93 (0.84, 1.03)		0.94 (0.81, 1.08)	0.38
Educational Status of the participants	Below primary	108 (80.6%)	42 (75.0%)	0.39	0.69 (0.33, 1.46)	0.32	1.12 (0.41, 3.09)	0.83
	Primary and above	26 (19.4%)	14 (25.0%)		Ref.		Ref.	
Education level of the female head of the household	Below primary	116 (86.6%)	47 (83.9%)	0.64	0.82 (0.34, 1.95)	0.62	0.58 (0.19, 1.82)	0.35
	Primary and above	18 (13.4%)	9 (16.1%)		Ref.		Ref.	
Family size	<10	116 (86.6%)	47 (83.9%)	0.13	1.61 (0.86, 3.02)	0.14	3.64 (1.56, 8.49)	<0.01
	≥10	18 (13.4%)	9 (16.1%)		Ref.		Ref.	
Income Quartiles	1st Quartile	38 (28.6%)	9 (16.1%)	0.32	0.54 (0.2, 1.395)	0.21	0.57 (0.16, 2)	0.38
	2nd Quartile	35 (26.3%)	17 (30.4%)		1.08 (0.46, 2.58)	0.87	0.95 (0.32, 2.84)	0.92
	3rd Quartile	31 (23.3%)	17 (30.4%)		1.21 (0.51, 2.93)	0.67	1.32 (0.45, 3.88)	0.61
	4th Quartile	29 (21.8%)	13 (23.2%)		Ref.		Ref.	
Biomarkers status	Serum Vitamin D	22.5 (15.7, 28.4)	21.9 (17.8, 25.3)	0.57	0.87 (0.41, 1.61)	0.68	0.48 (0.11, 1.99)	0.31
	Serum Ferritin	37.2 (26.0, 58.3)	42.0 (30.5, 63.7)	0.17	1.37 (0.93, 2.1)	0.12	1.55 (0.93, 2.57)	0.09
	Serum Vitamin B12	237.0 (179.0, 357.0)	165.0 (119.5, 255.0)	<0.001	0.31 (0.15, 0.58)	<0.001	0.29 (0.14, 0.62)	<0.01
	Serum Folate	5.0 (3.9, 6.7)	3.6 (2.7, 4.2)	<0.001	0.14 (0.06, 0.32)	<0.001	0.1 ( 0.03, 0.27)	<0.001

Abbreviation: Ref. = Reference cateogry.

## Data Availability

Not applicable.

## Supplementary Materials

The following supporting information can be downloaded at: https://www.mdpi.com/article/10.3390/nu14091751/s1, Figure S1: Homocysteine level distribution by gender.
