# Supplementary figures and images for: Adolescent Afghan Refugees Display a High Prevalence of Hyperhomocysteinemia and Associated Micronutrients Deficiencies Indicating an Enhanced Risk of Cardiovascular Disease in Later Life
Source: Nutrients. 2022 Apr 22;14(9):1751. doi: 10.3390/nu14091751 (PMC9105069; doi:10.3390/nu14091751)

## Supplementary Materials

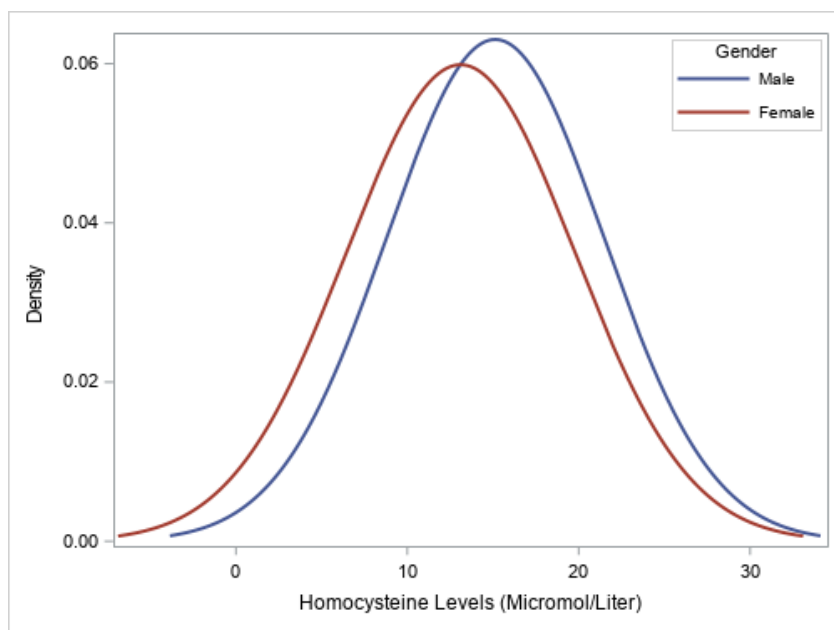

Figure S1: Homocysteine level distribution by gender

Supplement: Supplementary file 1 [file nutrients-14-01751-s001.zip › nutrients-1655168-supplementary.pdf]
